# Mechanisms Underlying Dysregulation of miR-132 in Alzheimer’s Disease

**DOI:** 10.26717/bjstr.2019.22.003824

**Published:** 2019-11-15

**Authors:** Qiaoyun Song, Juan Dou, Zixu Mao, Zhexing Wen, Wenming Li

**Affiliations:** 1 Department of Pharmacology and Chemical biology, Emory University School of Medicine, USA; 2 Department of Psychiatry and Behavioral Sciences, Emory University School of Medicine, USA; 3 Department of Reproductive Genetics, Hebei General Hospital, PR China

## Opinion

Alzheimer’s disease (AD), the most common format of dementia, is an increasingly prevalent and complex neurogenerative disorder in the elderly and among the leading causes of a miserable life quality and death worldly and characterized pathologically by both abnormal plaques consisting of aggregated amyloid β (Aβ) and neurofibrillary tangles of hyperphosphorylated tau [1]. microRNAs (miRNAs), small non-coding RNAs that regulate the translation of targeted mRNAs, are predicted to regulate up to 90% of the genes in humans, suggesting that they may control every cellular process in all cells and tissues of the human body [2]. Not surprisingly, alterations of individual miRNAs have been implicated in the AD pathological condition. The number of miRNAs is dysregulated in the AD disease conditions. Several neuroprotective miRNAs, especially miR-132, are downregulated in the AD patient brain, while several neurodegenerative miRNAs are upregulated in the same AD context [3]. Identifying what reasons and mechanisms cause the differentiative expressions of miRNAs may be key to the understanding of AD pathogenesis and the approaching of miRNAs for the AD therapy. Here we take miR-132 as an example to briefly discuss what mechanisms mediate its dysregulation under Alzheimer’s conditions.

Interplay of miR-132 with AD: miR-132 is specifically expressed and enriched in mammalian brains [4]. miR-132 and its paralogue miR-212 gene locates in human chromosome 17. Mature miR-132 sequences of 22 base pairs are processed from its precursor sequence of 66 nucleotides. Human miR-132 consists of two homologous miRNAs, i.e., miR-132–5p and miR-132–3p. The latter is a significant part of the miR-132/212 cluster [5]. Several lines of evidence show that miR-132–3p is downregulated with pathologic AD and associated with neuritic Aβ plaques and neurofibrillary tangle pathologies in AD brains [6, 7]. miR-212/132 deficiency in a mouse model leads to impaired memory, enhanced tau pathology, and excessed Aβ production/senile plaque formation as seen in AD patients. Mechanism study shows that miR-132 can target and downregulate tau, MAPK, sirt1, FOXO1a, ITPKB, PTEN, and FOXO3a, which are implicated in tau production, splicing, and phosphorylation [8–10], Aβ metabolism and deposition [8,9], or programmed neuronal death [11]. Interestingly, miR-132 is consistently upregulated in patients suffering from mild cognitive impairment (MCI), which may be benefit response to the AD-associated stress to maintain brain integrity in the early stage of disease [12]. Other researchers report that this miRNA drops in people with vascular dementia as well [13,14]. More importantly, miR-132 seems to protect against toxic Aβ and tau and to enhance the hippocampal long-term potentiation in both rodent models and human neurons [15]. To examine the neuroprotective properties of naturally occurring miRNAs, Dr. Krichevsky’s group has demonstrated that miR-132 is the most neuroprotective miRNA among 63 neuronal miRNAs [16]. Collectively, miR-132 has multiple neuroprotective activities, and its alteration aggravates multiple layers of AD pathology at the molecular and functional levels [6,8].

Potential mechanisms underlying miR-132 dysregulation in AD: The level of miR-132 depends on its biogenesis and degradation. miRNA biogenesis is controlled by several tightly coupled sequential steps. Most miRNAs are generated via the canonical pathway consisting of three tightly coupled steps [17]. miRNAs are initially transcribed from their genes by RNA polymerase II as the pri-miRNAs, which typically are over 1 kb and contain a terminal loop, a stem, and two flanking sides. Drosha and DGCR8 in the microprocessor complex process pri-miRNAs into the pre-miRNAs [17,18]. After transported into the cytoplasm by exportin-5, pre-miRNAs are further processed by the second RNase III enzyme Dicer to generate a double-stranded miRNA duplex of about 22 nucleotides [19]. Either strand of the duplex, i.e., miRNA-5p and miRNA-3p, is loaded into the RNA-induced silencing complex involving Argonaute family proteins to repress mRNA translation or reduce its stability [20,21]. miRNA biogenesis is highly regulated, but biogenetical processes and mechanisms underlying individual miRNAs, especially ones associated with AD, are poorly understood. For miR-132 under normal conditions, BDNF can enhance the transcription of pri-miR-132 possibly through the ERK1/2-MSK-CREB pathway [22].

However, no study answers which stage of miR-132 biogenesis is dysregulated under AD conditions. By measuring and analyzing the levels of pri-, pre-, and mature miR-132 under several AD conditions, we have found that the dysregulation of miRNA-132 occurs at the step from its pri- to pre-miRNA (unpublished data). We propose that the altered proteins in the pri-miR-132 microprocessor complex by AD stress deregulate its biogenesis and cause AD-associated pathological changes. To test this hypothesis, we have established a novel system to identify altered proteins in one specific individual miRNA microprocessor complex by using the unique pri-miRNA hairpin-based hybridization precipitation (RHP) technique. Several novel proteins have been identified as regulators for miR-132 biogenesis from the pri-miR-132 microprocessor under AD conditions (unpublished data), which may be involved in the mechanisms underlying miR-132 dysregulation under AD conditions although we cannot rule out the possibility of miR-132 dysregulation occurring at the stage of its degradation.

Although Biogen will pursue a regulatory approval for aducanumab because this antibody targeting amyloid in the Phase III EMERGE trial has met its primary endpoint, showing a significant decrease in clinical decline, miR-132 may still offer hope for the novel Alzheimer’s treatment [16], but we have to know how to target miR-132 for Alzheimer’s therapy because miR-132 is a multifaceted miRNA:

- More than a dozen targets for miR-132 have been identified beyond the central nervous system [23];
- Besides as the mediator to regulate neuronal differentiation and maturation and to participate in axon growth, neural migration, and plasticity, miR-132 is implicated in much non-neuronal functioning such as inflammation and angiogenesis [5,24]; and
- miR-132 may induce neuronal apoptosis and enhance tau phosphorylation under certain AD conditions [25]. Therefore, the investigation of mechanisms underlying alteration of miR-132 in AD may not only help us understand the AD pathogenesis but also provide promising therapeutic targets for AD.

Only understanding of the mechanisms underlying dysregulation of miR-132 can help us to correct such a dysregulation, which may serve as more effective therapeutic strategies to address and modify AD pathological processes.

## Abbreviations:

CREB: cAMP-Response Element Binding Protein
DGCR8: DiGeorge Critical Region 8
ERK: Extracellular Signal-Regulated Protein Kinases
FOXO: Forkhead Box O
ITPKB: Inositol-Trisphosphate 3-kinase B
MAPK: Mitogen-Activated Protein Kinase
MSK: Mitogen- and Stress-Activated kinases
